# Rapid and precise identification of cervicothoracic necrotizing fasciitis caused by *Prevotella* and *Streptococcus constellatus* by using Nanopore sequencing technology: a case report

**DOI:** 10.3389/fmed.2024.1447703

**Published:** 2024-10-21

**Authors:** Manna Zhao, Xuejun Leng, Jie Xu, Juanjuan Cui, Shuo Li, Weifeng Zhao

**Affiliations:** ^1^Department of Infectious Diseases, The First Affiliated Hospital of Soochow University, Suzhou, China; ^2^Infectious Diseases Department, Suzhou Kowloon Hospital, Shanghai Jiaotong University School of Medicine, Suzhou, China; ^3^Center of Clinical Laboratory, The First Affiliated Hospital of Soochow University, Suzhou, China; ^4^Key Laboratory of Digital Technology in Medical Diagnostics of Zhejiang Province, Dian Diagnostics Group Co., Ltd., Hangzhou, Zhejiang, China; ^5^Nanjing Dian Diagnostics Group Co.,Ltd., Nanjing, China

**Keywords:** cervicothoracic necrotizing fasciitis (CNF), necrotizing soft-tissue infections (NSTIs), nanopore sequencing technology (NST), coinfection, case report

## Abstract

**Introduction:**

Cervicothoracic necrotizing fasciitis (CNF) is one form of necrotizing soft-tissue infections, which could lead to patient demise during short course. Therefore, early recognition and immediate treatment contribute to promising prognosis of patients.

**Case presentation:**

A 58-year-old diabetic patient presented with a sore throat and progressive irritation of the neck and chest for 4 days. The initial diagnosis was considered to be soft-tissue infection and the clinician gave empirical anti-infectious medication for expectant treatment. During the course of disease, surgical incision was performed to relieve suffocation and shortness of breath. The drainage fluids were detected with microbiological culture and molecular sequencing. Nanopore sequencing technology (NST) helped to identify the coinfection of *Streptococcus constellatus* and *Prevotella* spp., which was not recognized during the original period of 15 days. The precise identification of pathogen supported to guide the pharmacologic treatment with meropenem and linezolid. Ultimately, combined with the surgical observation and post-surgical pathological examination, the patient was diagnosed as CNF, which could be much more acute and serious than normal soft-tissue infections. The patient has been successfully treated with prompt antimicrobial medication and appropriate surgical debridement.

**Conclusion:**

This case presented a CNF patient with type 2 diabetes, successfully recovered after prompt microbial detection, precise anti-infectious treatment, and appropriate surgical intervention. It highlights the importance of recognizing pathogen by applying rapid microbiological detection, including NST, in acute and serious infectious disease.

## 1 Introduction

Necrotizing soft-tissue infections (NSTIs) is a deep soft-tissue infection causing progressive destruction of muscle fascia and overlying subcutaneous fat (1), with the incidence of 0.3 to 15 cases per 100,000 population, but a mortality of over 20% (2). Cervicothoracic necrotizing fasciitis (CNF) is one form of NSTIs with relatively rare infections of head and neck, compared with other body areas reported, and could lead to patient demise during short course (3). Thus, early recognition and immediate treatment are key to favorable outcomes of CNF patients.

The definitive diagnosis of necrotizing fasciitis relies on the surgical evaluation, and pharmacologic treatment is recommended being guided with microbiological identification (4), especially Gram’s staining (1, 5). Traditional microbiological detection includes Gram staining, culture and antimicrobial sensitivity information (1), which could be time-consuming (6) and coverage-restricted (7). Nanopore sequencing technology (NST) is an emerging platform for pathogenic molecular detection, which is a promising method for fast, precised, and broad-range microbial identification (8, 9). Therefore, NST might contribute to the improvement of prognosis in patients with necrotizing fasciitis.

Herein, we presented a successfully treated CNF patient with type 2 diabetes. Guided with NST, the clinician identified the coinfection of pathogenic *Prevotella* and *Streptococcus constellatus*. With the combination of surgical debridement and prompt antimicrobial treatment, the patient achieved clinical cure and discharged after 46-day treatment. To our knowledge, cases of CNF identified with bacterial coinfection by NST have been rarely reported. It suggests that NST could provide rapid and precised clue of anti-infectious medication, and be benefit on CNF diagnosis and treatment.

## 2 Case presentation

**Figure 1:**
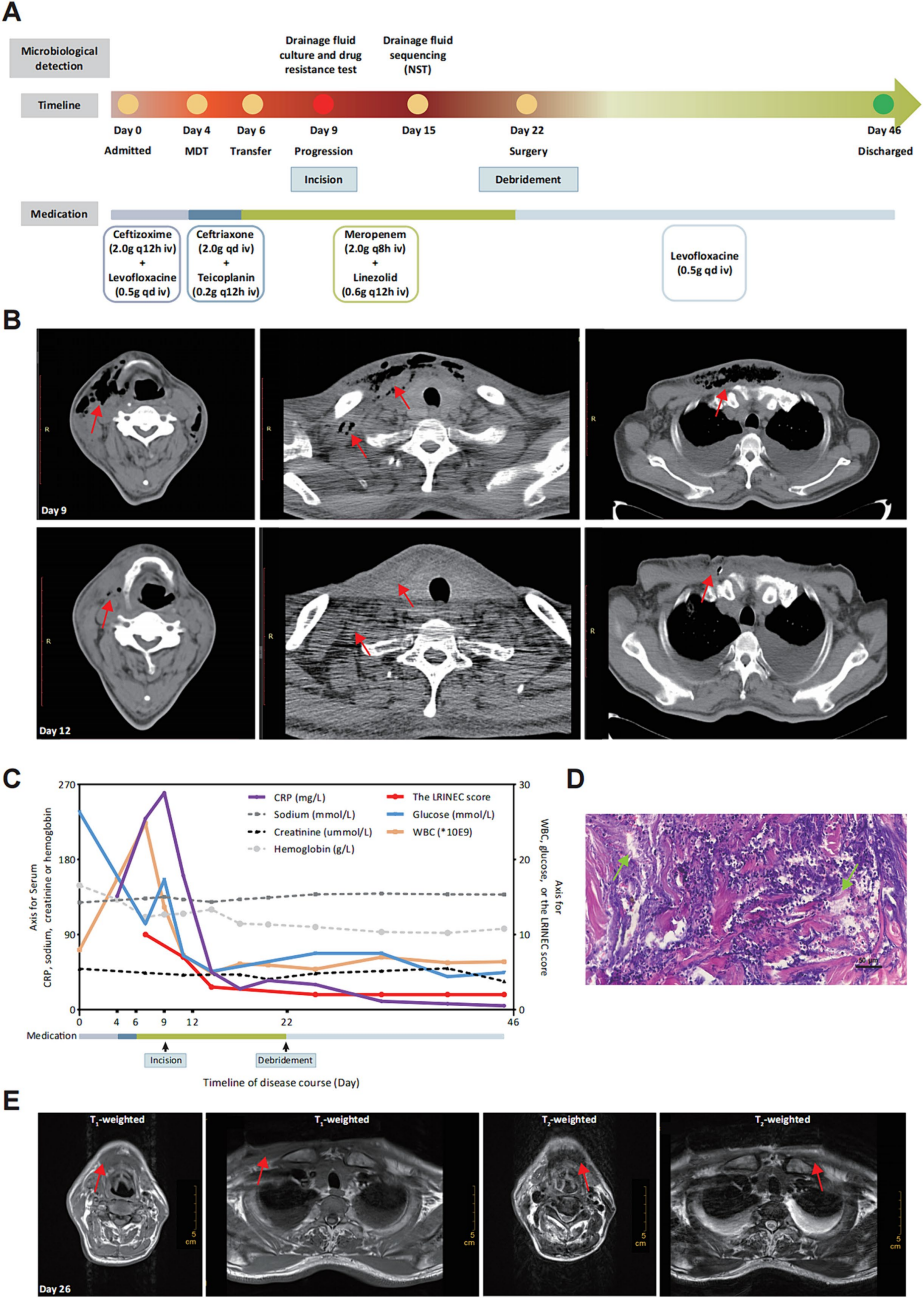
Process and diagnosis of CNF case. **(A)** Timeline of disease detection, diagnosis and treatment. **(B)** Image morphology of cervical and chest CT scan before (Day 9) and after (Day 12) thoracentesis incision. Disease progression at Day 9 and symptoms improvement after incision at Day 12. The subcutaneous emphysema was decreased significantly with bilateral pleural effusion (marked with red arrays). **(C)** Changing trends of clinical indicators and the LRINEC score retrospectively calculated during the course of disease. WBC, white blood cell; CRP, C-reactive protein. **(D)** Pathological examination exhibited tissue abscess and necrosis (marked with green arrays) with hematoxylin and eosin (H&E) staining (10 × 20). **(E)** Image morphology of cervical and chest MRI (Day 26). There is no significant subcutaneous emphysema 4 days after surgical debridement (marked with red arrays).

A 58-year-old male patient presented to the clinic with a chief complaint of sore throat for 4 days. The pain was particularly noticeable while swallowing, accompanied with a hoarse voice. Despite receiving anti-inflammatory treatment, the sore throat was not ameliorated significantly. As a result, the patient was admitted to the Otorhinolaryngologic Department (Day 0). Upon admission, the patient’s vital signs were as follows: ear temperature of 37.4°C, heart rate of 102 bpm/min, blood pressure of 98/59 mmHg, and respiratory rate of 21 times/min. The clinician gave empirical treatment with ceftizoxime and levofloxacin (Figure 1A).

At Day 4, the patient began experiencing progressive irritation in the neck and chest. The dermathemia and swelling of cervicothoracic skin was obvious, with a high skin temperature, obvious skin tenderness, and palpable skin fluctuation and crepitus. Following multidisciplinary consultation, the initial diagnosis was considered to be soft-tissue infection, and the medication were empirically changed as ceftriaxone and teicoplanin. With no significant improvement of the chief complaints, the patient was transferred to the Infectious Department for infectious control and further treatment (Day 6). Given the high level of inflammatory indicators and the unsatisfactory response to conventional antibiotics, the medication was switched to meropenem and linezolid. It was worth noting that the patient was diagnosed with type 2 diabetes mellitus (T2DM) during hospitalization. For the purpose of further diagnosis, the cervical and chest computed tomography (CT)-scan was performed at Day 9, which showed swelling and pneumatosis in the right parts of vocal cord, parapharyngeal space and cervicothoracic subcutaneous tissue (Figure 1B), with inflammatory changes. However, the disease suddenly progressed with the chief complaints being increased suffocation and shortness of breath in the afternoon of Day 9. To relieve discomfort, thoracentesis was performed, along with skin incision and effusion drainage. The drainage fluid was positively cultured with colonies of *Streptococcus mitis*, which was covered with the anti-infectious spectrum of present medication. Three days later (Day 12), the symptoms of swelling and pneumatosis exhibited significant amelioration, according to the CT-scan (Figure 1B, Day 12). For a comprehensive and accurate pathogen diagnosis, molecular sequencing with NST of drainage fluid was performed, which revealed positive detection for *Prevotella intermedia*, *Prevotella enoeca* and *Streptococcus constellatus* (Table 1). The NST result suggested polymicrobial infection, which was not recognized during the last 15-day treatment. Fortunately, the spectrum of anti-infectious drugs could cover the detected pathogen. The level of inflammation biomarkers, monitored in the duration of therapy, continued to decrease (Figure 1C), indicating the effectiveness of anti-infection treatment.

**Table 1:** Types of pathogens detected by metagenomic third-generation sequencing.

Genus	Species	Number of detected reads
*Prevotella*	*Prevotella intermedia*	773
*Prevotella*	*Prevotella enoeca*	317
*Streptococcus*	*Streptococcus constellatus*	252

Despite the improvement of clinical indicators (Figure 1C) and imaging features, the patient still complained about the discomfort of cervicothoracic tension and pain. Thus, the diagnosis suspected to be CNF and thoracotomy debridement is recommended (1) and performed (Day 22). Extensive liquefaction of subcutaneous tissue and part of deep fascia was observed during the operation and the pathological examination was consistent with tissue abscess and necrosis (Figure 1D), which both confirmed the diagnosis of CNF. Laboratory Risk Indicator Score for Necrotizing Fasciitis (LRINEC) score is a tool to distinguish CNF from other soft tissue infections (10). We retrospectively calculated the LRINEC scores during the course of disease, which exhibited a gradual downward trend (Figure 1C). Moreover, after confirming the diagnosis of CNF, magnetic resonance imaging (MRI) was performed at Day 26. Results of MRI exhibited the shrinkage of soft-tissue lesion and amelioration of the CNF (Figure 1E). Evidences mentioned above further confirmed the effectiveness of our treatment. Ultimately, the patient recovered uneventfully from the surgery and discharged from hospital on Day 46.

## 3 Discussion

This case presented a diabetic patient diagnosed as CNF and successfully treated. With rapid detection of microbial sequencing based on NST, coinfection of *Prevotella* and *S. constellatus* was additionally identified, which contributed to the disease recognition and therapeutic success. Characterized with rapid progression and high mortality, CNF could exhibit improved prognosis with prompt recognition, appropriate surgical debridement, and precise antimicrobial medication. Molecular detection, including NST, could provide precise data on pathogenic microorganisms, which is essential for promptly adjusting antibiotic therapy for progressive diseases, such as CNF.

NSTIs is generally divided into four types, according to epidemiological features and causes (1, 11). Type I NSTIs is characterized by polymicrobial infection, usually includes both aerobic and anaerobic bacteria. Type II NSTIs is commonly monomicrobial infection, especially Gram-positive organisms, such as group A Streptococcal (GAS) and methicillin-resistant *Staphylococcus aureus* (MRSA) (12). Infections caused by other pathogenic microbes, such as Gram-negative microbes and fungi, are classified as type III and Type IV NSTIs, respectively. The causative microorganism of this case was cultured as *S. mitis* only, and it suspected to be type II NSTIs. With Nanopore sequencing, pathogenic microorganisms were identified as *Prevotella* spp. and *Streptococcus* spp. Combined with elderly age and diabetes, the true classification should be type I NSTIs for this patient. Besides, clinical routine culture is usually aerobe-covered only and consuming 2 to 7 days. In contrast, Nanopore sequencing as an emerging platform, carries broader microbial coverage and shorter time of detection (9), which significantly contributes to type identification based on epidemiological features and causes, leading to a better understanding of NSTIs and control of disease progress.

Clinical manifestation plays a crucial role in the management of CNF. Unfortunately, during the initial phase, symptoms of CNF May be subtle or even absent. Combined with absence of specific laboratory indicators (13), those could lead to delayed treatment for CNF patients (14). CT scan and MRI are sufficient assessment for CNF. Nevertheless, surgical exploration remains the only definitive method for diagnosing NSTIs (15). Previous reported cases of CNF would emphasize the importance of surgical intervention on CNF treatment, especially for case with synchronous CNF and pharyngocutaneous fistula (16), patient with acute epiglottitis and abscess complicated with CNF (17), and surgical reconstruction of CNF (18). Combined with surgical debridement, control of hyperglycemia and source of infection would significantly affect the outcome of CNF (19). Thus, timely diagnosis and supportive therapies, including anti-infective drugs, surgical exploration and debridement, are crucial for improving patient outcomes. A case reported a patient initially treated as deep neck infection while later found descending necrotizing mediastinitis with CNF. The patient underwent repeat surgeries within the overall duration of 220 days (20). For this case, we used Nanopore sequencing for precise pathogen identification, supplement to the initial detection of culture and Gram staining. Results of NST supported the definitive antibiotics medication before and after the surgical debridement, fortunately achieving successful treatment of this high-risk case within 46 days.

*Prevotella* is a Gram-negative bacteria and *S. constellatus* is a Gram-positive coccobacillus, both of which are anaerobic and present in the oral microbiome of healthy people (21). There have been a few reported cases where certain strains of *Prevotella* and *S. constellatus* have caused opportunistic endogenous infections (22, 23), such as chronic infections, abscesses, and anaerobic pneumonia (21, 24). Although rarely reported, *Prevotella* and *S. constellatus* has been associated with NSTIs in recent case reports (25, 26), and found present in all NSTIs cases of fatality (27). For the patient of this case, considering the initial symptom of a sore throat and the presence of conditional pathogenic *Prevotella* and *S. constellatus* in the normal oral cavity, it is highly likely that the pathogenic bacteria originated from the oral cavity. Furthermore, the identification of *Prevotella* should not be underestimated in NSTIs cases.

Pathogen detection is an important component in the diagnosis and treatment of infectious diseases. Nanopore platform is capable of direct RNA and DNA sequencing, providing a theoretical advantage in clinical diagnostics on both rapid species identification and antimicrobial resistance gene detection (9, 28). Cases of microbial identification and management of CNF patients were also previously reported, while the methodology was mainly utilized with culture (29, 30). In this particular case, traditional microbial culture took at least 72 h to identify a positive coccus with unexplained subcutaneous emphysema, whereas NST successfully identified the presence of *Prevotella* and *S. constellatus* infections. Lydia-Ann J Ghuneim et al. has demonstrated that the final outcomes of antimicrobial therapy to polymicrobial infections are dictated by the complex microbial ecology, and the precised identification of ecological members May optimize antimicrobial treatment (31). Studies have proven that high-throughout sequencing, such as metagenomic next-generation sequencing, could improve detection rate for pathogen in polymicrobial bloodstream infections (32), polymicrobial brain abscess (33), and complex pleural infections (34). The results of this study provide supportive evidences for excellent performances of NST in pathogen detection. It highlights the usefulness of NST in identifying pathogens that are challenging to grow by using conventional methods. The prognosis could be significantly enhanced by early diagnosis using NST, identification of the causative pathogens, and targeted administration of antibiotic therapy.

## 4 Conclusion

In conclusion, this case presented an elderly high-risk CNF patient with type 2 diabetes, successfully recovered after prompt antibiotic treatment and appropriate surgical intervention. The key to this successful treatment relies on the appropriate application of molecular detection of NST for pathogen identification, precise anti-infective treatment, and collaborative efforts of surgical and multidisciplinary teams. NST is of great importance for the detection of unknown pathogens, and we expect further application of advanced sequencing technologies in acute and serious infectious diseases in the future.

## Data Availability

The datasets presented in this study can be found in online repositories. The names of the repository/repositories and accession number(s) can be found at: https://db.cngb.org/cnsa/, CNP0004703.
